# Pseudothrombocytopenia Inducing Nonindicated Platelet Transfusion after Cardiac Surgery

**DOI:** 10.1155/2021/3695407

**Published:** 2021-03-05

**Authors:** Enise Ceran, Christine Schlömmer, Ivonne Kröckel, Georg Scheriau, Philipp Angleitner, Barbara Steinlechner

**Affiliations:** ^1^Department of Surgery, Division of Cardiac Surgery, Medical University of Vienna, Vienna, Austria; ^2^Department of Anesthesia, Critical Care and Pain Medicine, Division of Cardiothoracic and Vascular Anesthesia and Intensive Care Medicine, Medical University of Vienna, Vienna, Austria

## Abstract

Pseudothrombocytopenia (PTCP) is an in vitro phenomenon of low platelet count caused by the agglutination of platelets, leading to false low platelet counts in automated cell counting. Typically, ethylenediaminetetraacetic acid (EDTA) mediates this platelet clumping. PTCP has little clinical significance, but misdiagnosis may lead to unnecessary diagnostic tests and treatment. In this case report, we present a 65-year-old Caucasian female suffering from multiple complications during and after cardiac surgery. During her postoperative stay at the ICU, she was diagnosed with thrombocytopenia and an inadequate response to platelet supplementation.

## 1. Case Presentation

**Figure 1:**
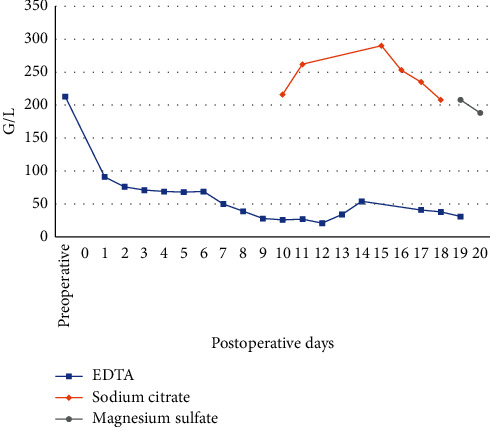
Postoperative platelet count with different blood collection tubes (EDTA, Sodium Citrate, Magensium sulfate) as shown.

**Figure 2:**
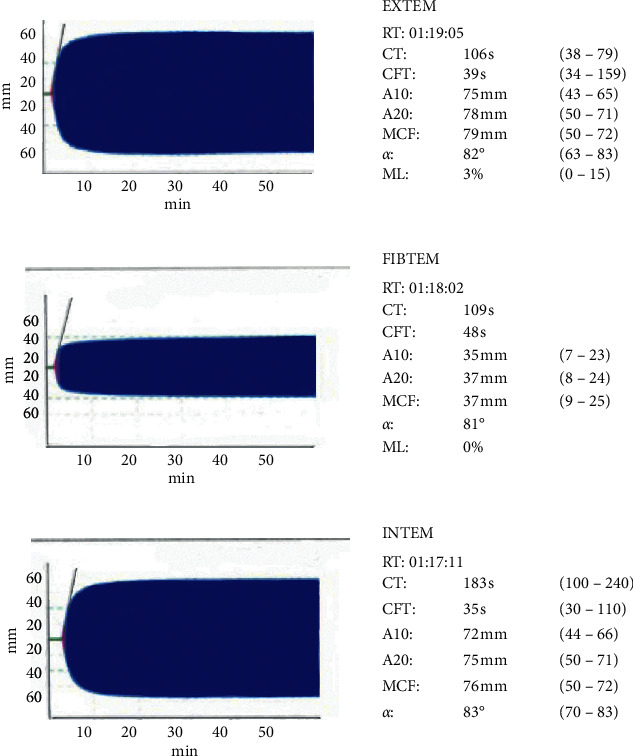
Rotational thromboelastometry (ROTEM) showing a procoagulant state despite the low numeric platelet count.

A 65-year-old Caucasian female was admitted to the General Hospital of Vienna with the diagnosis of a combined high-grade mitral and tricuspid valve insufficiency, persisting atrial fibrillation, and a hypertrophic nonobstructive cardiomyopathy. Surgical reconstruction of the mitral and tricuspid valve, as well as a left atrial appendage (LAA) resection, was indicated and performed. An intraoperative rupture of the left atrium led to two periods of cardiopulmonary bypass and the consecutive implantation of venoarterial extracorporeal membrane oxygenation (ECMO) due to impaired right ventricular function. After the surgical procedure, the patient was transferred to the ICU, where she required high doses of catecholamine support and continuous venovenous hemodialysis (CVVHD) due to acute renal failure. On postoperative day (POD) 7, ECMO weaning was successful, and the therapy was finally stopped. Initially, anticoagulation therapy consisted of unfractionated heparin and was switched to low-molecular-weight heparin after ECMO explantation. On POD 8, a complete blood count (CBC) showed a reduced platelet count of 39 G/L (Figure 1). 4Ts score for heparin-induced thrombocytopenia (HIT) was 5, which represented an intermediate probability of HIT. An enzyme-linked immunosorbent assay (ELISA) for HIT was negative (Zymutest HIA IgG, Hyphen Biomed, Neuville sur Oise, France), and a viscoelastic test of a whole blood sample (rotational thromboelastometry, ROTEM) showed no abnormalities, moreover, a procoagulant state despite the low numeric platelet count (Figure 2). Physical examination did not reveal any signs of hemorrhagic diathesis. Platelet count could not be significantly increased by the transfusion of two units of platelet concentrates. The platelet count after the first transfusion was 28 G/L, and the platelet count after the second transfusion on the following day was 24 G/L. On POD 10, CBC was reevaluated by using sodium citrate tubes instead of EDTA tubes, revealing a platelet count of 216 G/L (Figure 2). Since PTCP was diagnosed two days after the first low platelet count and the patient was critically ill and on continuous ECMO therapy, other causes of thrombocytopenia such as immune thrombocytopenia were not evaluated further. During the patient's stay at our ICU, platelet count was also checked with a specific blood collection tube (ThromboExact, Sarstedt, Nümbrecht, Germany) confirming the results collected with the citrated blood tubes.

## 2. Discussion

Thrombocytopenia is defined as a platelet count lower than 150 G/L. A platelet count lower than 100 G/L is considered to imply medium to high bleeding risk, depending on the particular platelet count [1]. The diagnosis of thrombocytopenia usually includes a repetition of the complete blood count [2–4], the white blood cell differential [5], a peripheral blood smear, and the evaluation of possible hematological abnormalities. If none of these tests are indicative for a genuine thrombocytopenia or a malignant hematological disease, PTCP can be the cause for low platelet count [6, 7].

PTCP is an analytical error in automated blood cell counting. Blood samples which contain autoantibodies against thrombocytes and are collected in EDTA-containing tubes can lead to platelet clumping. Agglutinated platelets are detected as larger cells by using automated counters and lead to false low results. This phenomenon was discovered early after the invention of laboratory medicine automation and was described precisely soon after [8].

The incidence of severe thrombocytopenia (<100 G/L) among the population of critically ill patients is 7.8% and therefore a very likely diagnosis. This could mislead clinicians in cases of PTCP and result in overtreatment with the administration of platelet concentrates or even more invasive procedures such as splenectomy. Other rare causes of thrombocytopenia such as HIT were found to be 0.3% in this population [9], whereas the incidence of PTCP is suggested to range from 0.09 to 0.15% in routine clinical blood samples [10, 11]. Unfortunately, data of PTCP in a critically ill population are lacking, but given the data, one can assume that the likelihood of other more clinically relevant causes of thrombocytopenia, even if it is still very small (HIT 0.3%), is higher than to suffer from PTCP. Therefore, clinicians are concerned with the diagnostics of the more severe forms of thrombocytopenia and are not aware about analytical errors since the repetition of the specimen often confirms the result.

As mentioned above, PTCP is induced by the cross-reaction of EDTA and platelet autoantibodies which cause the agglutination of platelets in the sample [8]. This phenomenon is not only restricted to EDTA but also present with other anticoagulants such as sodium oxalate, sodium citrate, and heparin, making it more difficult to diagnose PTCP [12]. Recently, there are increasing numbers of reports portraying this multi-anticoagulant PTCP [13–15]. Additives mixed with EDTA specimens, i.e., aminoglycosides such as kanamycin and amikacin, can prevent the agglutination of platelets and dissociate platelet clumps if added after blood withdrawal and could therefore be a helping agent in diagnostics [16, 17]. Unfortunately, there have been reports on the unreliability of these effects in certain cases of PTCP [11]. Another possible solution of the problem could be the utilization of magnesium sulfate (MgSO_4_) as an anticoagulant. Before laboratory medicine automation, MgSO_4_ was widely used to prevent blood cell agglutination but was replaced by EDTA after the invention of automated cell counting because of the more convenient use as spray-dried coat in blood collection tubes. It is now suggested to reconsider MgSO_4_ as an anticoagulant in diagnostics of multiple-anticoagulant PTCP [18]. By now, blood collection tubes containing MgSO_4_ as an anticoagulant additive are available commercially (ThromboExact, Sarstedt, Nümbrecht, Germany) and were used in this presented case to confirm the results, which were collected in citrated blood tubes.

The induction of autoantibodies reacting with EDTA and platelets is not resolved but very likely induced over a period of medical treatment. An early work on the patient population (18 patients) presenting with PTCP found that, at hospital admission, 61% of all patients had a platelet count over 100 G/L, which then deteriorated over their hospital stay. Furthermore, they found that 55% of the study population were treated at an intensive care unit, 22% suffered from neoplastic diseases, and another 22% suffered from atherosclerosis-related conditions [19]. However, newer investigations could not reveal a correlation between these clinical characteristics and the development of PTCP, except for receiving medical treatment within a short period of time [10, 11].

The assumption of antibodies being the cause of this phenomenon was communicated early after the introduction of PTCP [8]. The detection of specific antibodies took place in the mid-90s. Platelet agglutination is induced by a variety of immunoglobulins including IgA, IgG, and IgM antibodies, which bind to glycoprotein IIb/IIIa receptors on the platelet surface [20].

There are growing numbers of reported PTCP caused by a variety of therapies and diseases. In some cases, immunotherapy with immune checkpoint inhibitors seems to be responsible for PTCP [21], as well as direct glycoprotein IIb/IIIa antagonists such as abciximab [22]. Furthermore, there have been reports on PTCP in COVID-19 patients [23]. It seems that multiple medical treatments, diseases, and infections can induce PTCP.

It is therefore important to raise awareness throughout the clinical and scientific societies to consider PTCP as differential diagnosis early after the onset of low platelet counts without clinical symptoms of hemorrhagic diathesis. With cases of multi-anticoagulant PTCP, it is getting more difficult to detect false low platelet counts, and appropriate diagnostic facilities (peripheral blood smear, ThromboExact blood tubes) need to be available for correct diagnosis. Considering these challenges, it can be difficult in clinical settings to thoroughly evaluate causes of thrombocytopenia, but one should consequently pursue the process of finding the right diagnosis since the clinical consequences of overtreatment can be dramatic, i.e., splenectomy. In this case report, the challenge of finding the right diagnosis was the complexity of the case. Thrombocytopenia is not uncommon after cardiac surgery and on ECMO therapy. A bleeding tendency is often present because of deranged coagulation factors and mass transfusions. Since our department is very skilled and trained in the management and diagnosis of bleeding disorders, our patient was diagnosed with PTCP shortly after the onset of severe thrombocytopenia (two days after first severe low platelet counts). Further invasive treatment could be averted from the patient.
